# Analysis of Human Behavior for Robot Design and Control

**DOI:** 10.1155/2020/1726347

**Published:** 2020-02-04

**Authors:** Francesca Cordella, Loredana Zollo, Michelle J. Johnson

**Affiliations:** ^1^Unit of Advanced Robotics and Human-Centred Technologies, Campus Bio-Medico University of Rome, Rome, Italy; ^2^Department of Physical Medicine and Rehabilitation, University of Pennsylvania, Philadelphia, USA

Advances in biologically inspired robotic systems that is aimed at reproducing mechanics, control, sensory and actuation systems of human beings are rapidly growing, especially in the fields of human-robot interaction and cooperation. Indeed, studying and replicating the human behavior can provide new insights into the development of robotic and mechatronic systems operating in the medical domain (rehabilitation, assistance, and surgery) and, more in general, in all the application domains where collaborative robotics is being applied.

In this special issue, we present several challenging aspects of analysis and modeling of human behavior in robotics with special attention to a key aspect, i.e., how modeling and analysis can be translated into concrete guidelines for robot design and control. The focus is on novel systems and methods for the observation and analysis of human behavior and advanced approaches to replicate this behavior in robotic systems conceived for human-robot interaction. In particular, this special issue will provide an evidence of the paramount role of human behavior analysis and, in general, human inspiration in the following domains: robot teleoperation, design and control of robotic hands for prostheses, development of patient-tailored rehabilitation protocols, and design and control of assistive and rehabilitation devices (e.g., lower-limb rehabilitation devices, exoskeletons, and head and neck positioning devices).

The paper by Rybarczyk et al. shows the intuitiveness of human control of a bioinspired motion pattern with respect to nonbioinspired control. The quality of the user control of a mobile robot was assessed by comparing teleoperated control of the bioinspired motion pattern where the robot speed is set according to the two-third power law—a law that underlies human-like motion behavior—with the manual steering mode where speed and direction is under the users' control and the nonbioinspired steering mode where speed is set linearly with direction. The results demonstrated that the performance in terms of time required to complete the path, number of collisions with obstacles, and trajectory smoothness are significantly better in the biological condition than in the two other conditions. Therefore, implementing a human-like behavior on a robot could significantly simplify the ability of a human operator to control it.

Llop-Harillo et al. present an index that evaluates the anthropomorphism of artificial robotic hands for amputees by comparing the dexterity of artificial hands with the human hand. The index was derived by studying human grasping actions, functionality of human hands, and the components of robotic hands. Thirteen prosthetic robot hands are evaluated. Results obtained by the comparison of the proposed index with other metric indexes showed that the index is quick, appropriate, and efficient in evaluating the anthropomorphism of artificial robotic hands. Therefore, it could represent a useful tool in the hand design stage to maximize hand functionality.

The paper by Hu et al. proposes to explore a new method to enable faster robot learning from humans. The study uses a hierarchical learning from demonstration structure of task-parameterized models for everyday object movement tasks. The approach uses the task-parameterized Gaussian mixture model (TP-GMM) algorithm to encode sets of demonstrations in separate models each corresponding to a different task. It is shown that the approach produces better results as compared with learning a single TP-GMM model and allows the robot to generalize movements to undemonstrated ones. The results have been validated both in simulations and on real hardware.

Goffredo et al. analyzed kinematic data registered on 68 poststroke patients during 20 daily sessions of robot-aided upper-limb rehabilitation. A planar end-effector robot was used, and motor performance was evaluated during point-to-point trajectories executed with different direction changes. The obtained results, in terms of movement accuracy, movement, speed, number of peak speed, and task completion time, demonstrated an improvement in motor performance dependent on movement direction and the level of motor impairment. Their work suggests that rapid changes in kinematic data can be seen over the first 5 sessions and may be maximum for movements involving the shoulder and elbow flexion and extension. This outlines the importance of designing patient-tailored rehabilitative protocols.

In Ramakrishnan et al., traditional approaches to gait parameter analysis are examined to give a more comprehensive and broader perspective of gait. In particular, a new combined gait asymmetry metric (CGAM) was developed using 13 spatial, temporal, and kinetic gait parameters. The new index was used to evaluate how rehabilitation therapies change gait asymmetries on six poststroke patients. The experimental results showed that the proposed CGAM metric has the potential to be used as a quantitative metric for impairments causing gait asymmetries, where a decrease in the CGAM score after therapy means that subject gait improved.

The work by Wang et al. explores how human gait during overground walking on different grounds (i.e., different kind of pavements) affect interjoint coordination (knee-ankle and hip-knee) with and without exoskeleton. Experimental tests were performed on 8 healthy subjects and the continuous relative phase (CRP) has been analyzed to examine changes in kinematics across one gait cycle. The obtained results revealed that CRP is independent of whether or not the exoskeleton is used but varies depending on the compressive capacity and unevenness of pavements. CRP is a promising parameter for use in optimizing the design of exoskeletons for motor recovery and ambulation in real-world environments.

In Park et al.'s study, human gait during stair walking is explored to improve the design of gait training robots. The study highlights the importance of stair walk training. By analyzing stair gait patterns of 6 healthy subjects and using the obtained results, in terms of angular trajectories, joint range of motion, and joint relative displacement, they extract standard patterns of stair ascent and descent to be applied on a lower-limb robotic rehabilitation system for vertical motion of footplates. The resulting trajectories enabling natural stair-climbing motions.

In the paper by Miyake et al., human gait is again studied to improve exoskeleton design and the control of overground walking in exoskeletons. The study proposes a new method based on a radial basis function network to predict toe clearance during walking, with the aim of decreasing the risk of tripping. The authors measured several parameters of the hip, knee, and ankle joints of 11 subjects (data from 6 subjects were used for training the network and data from 5 subjects were used for testing the approach) at the beginning of the swing phase. The obtained results demonstrated that the proposed approach can be used to predict both the maximum toe clearance in the earlier swing phase and the minimum toe clearance in the later swing phase at the same time.

Finally, the paper by Zhang et al. explores human head and neck kinematics for the critical cancer treatment. They present a novel design for a proton heavy ion radiotherapy chair with a head and neck positioning device. The design is based on kinematic analysis of posture and on ergonomic evaluations. The proposed design was tested on 12 healthy subjects and the obtained results demonstrated that the proposed device meets the head and neck positioning requirements of users proving that a human-centred design is useful.

